# Resection of the Superior Maxillary

**Published:** 1858-03

**Authors:** W. F. Westmoreland

**Affiliations:** Prof. of Surgery in the Atlanta Medical College, Atlanta, Georgia


### ARTICLE. III.

*Resection of the Superior Maxillary.* By W. F. WESTMORELAND, M. D., Prof. of Surgery in the Atlanta Medical College, Atlanta, Georgia.

Betsey, aged 17 years, good constitution, the property of Mr. M——, of Fairburn, Ga., entered my Infirmary the first of October, 1857. In February preceding her entry, she was attacked with pain in the superior maxillary, which was regarded, at the time, as toothache, and one or two molars extracted; the pain, however, continued to increase, and with it the jaw increased in size, particularly in the region of the alveolar processes. This state continued for several months without any great change in the size of the tumor—the pain being paroxysmal. A few weeks, however, before she was placed under my care, the disease made rapid progress—the alveolar process at one point becoming exposed, presenting the appearance of caries of the bone.

Upon her arrival at the Infirmary, I found a considerable enlargement over the region of the left superior maxillary. The gums were greatly thickened, lobulated, hard and elastic, at some points projecting half an inch or more over the remaining teeth—completely enveloping them. They were, to some extent, vascular, but did not present the appearance usually observed in encephaloid disease.

The disease extended from the second incisor to the last molar. The denuded point of the alveolar process before alluded to, corresponded with the second molar, which had been extracted, this being the only point that presented any of the characteristics of malignant disease, and this, as above suggested, presenting more the appearance of simple caries than encephaloid degeneration.

Regarding the disease as Epulis, which had, or was likely to assume the malignant form of this disease, I decided to operate at once—proposing to remove only the diseased gum and alveolar processes.

From circumstances unavoidable, the operation was deferred for eight or ten days, in which time there was a marked increase in the size of the tumor, with an aggravation of all the symptoms.

On the 20th Oct., the operation was performed as follows:

With the patient half reclining, and the head well supported, an incision was made through the thickness of the cheek, from the left angle of the mouth to near the temporal fossa, and so directed as to leave the duct of the parotid gland with the external or inferior flap, uninjured. The nasal flap was now dissected up, leaving the diseased mass fully exposed. After the excision of the thickened and nodulated gum, and the extraction of all the teeth upon that side except the two incisors, I found the remaining tumor hard and non-elastic—presenting the appearance, in a striking manner, of an enlargement of the superior maxillary itself. By means of a strong pair of bone forceps, it was at once made evident that the upper jaw was not involved in the tumor — that the enlargement was entirely a new formation. With the chisel and mallet the tumor was removed without the least difficulty, it peeling off from the superior maxillary as readily as the bark from the oak—leaving this bone, if we except a slight caries before alluded to, to all appearance, perfectly sound; the only change being the absorption, to a considerable extent, of the alveolar processes. The mass thus removed was from half an inch to an inch and a half thick, and extended over a considerable portion of the superior maxillary. It consisted of a fibro-cartilaginous substance with an osseous deposit—the deposit being greatest at its base, or the portion in contact with the bone. Under the microscope it presented very much the appearance of a cartilage undergoing the process of ossification.

The operation was now completed by removing, with a strong pair of bone nippers, the entire alveolar processes.

The edges of the incision were brought together by means of a number of small silver pins and figure of 8 suture; and supported by means of two or three strips of adhesive plaster. The cold water dressing was kept constantly applied. The greater portion of the wound healed by the first intention. No unpleasant symptoms. The patient was up in ten or twelve days, and in three weeks was, to all appearances, well.

About five weeks after the operation, the pain in the jaw returned, and in a very few days there appeared at the point occupied by the second molar, or near that point, a fungus looking mass which rapidly increased in size. At the same time, there appeared in the roof of the mouth a slight tumor or projection, evidently the result of an extension of the disease to the antrum highmorianum. The disease made rapid progress, and at the expiration of eight or ten days there was a very great enlargement over the region of this bone. The patient for some time was not disposed to submit to a second operation, but after several day's intense suffering, gave her consent. The operation proposed, was the exsection of the entire superior maxillary with the greater portion of the malar.

On the 15th December, the second operation was performed, with the patient the greater portion of the time under the influence of chloroform. The position of the patient was the same as in the first operation—half reclining, with the head well supported, and so arranged, that its position could be changed at will. A curved incision was now made, commencing at the left commissure of the lips, and extending to a point over the temporal fossa, midway between the external angle of the eye and the external ear. In making the incision, the vessels were secured as soon as severed, and a piece of sponge kept constantly in the mouth to absorb the blood, which would have otherwise interfered with respiration—the patient at the time being under the influence of chloroform. The nasal or internal flap was now dissected up to the middle of the nose, cutting off the attachment of the cartilages of the left alœ of the nose; the dissection was also extended to the orbit, detaching the eye ball from the inferior portion of the orbit. More than once during this dissection, the blood flowed in torrents, and when possible, was arrested by ligating the vessels. It was sometimes, however, impossible to secure the vessels, the hemorrhage proceeding from numerous small arteries and veins, and was then readily arrested by the application of the perchloride of iron by means of a piece of sponge. A chain saw was next passed beneath the zygomatic process of the malar bone, detaching it without difficulty. With Hey's saw, a section of the frontal process of the malar bone was made just below the external canthus, and with a chisel and mallet the nasal process of the superior maxillary was severed from its connections with the nasal and frontal bones. With a strong scalpel the mucous membrane of the roof of the mouth was now divided over the median line, and the bone severed from its fellow of the opposite side, with Liston's bone forceps—it was afterwards further detached with the chisel and mallet.

It only remained, now, to divide the orbital plate. With the nasal flap tightly drawn back over the forehead, and the eyeball displaced upwards, the orbital surface was exposed, and severed with the chisel and mallet—dividing at the same time the infra-orbital nerve. The chisel was directed obliquely downward and backwards, and after passing through the orbital plate, was driven down between the upper jaw and the pterygoid process of the sphenoid bone, fracturing to some extent, the palate bone. After detaching all the soft parts that could be conveniently reached, the bone was started from its place, by making a lever of the chisel, and a fulcrum of the pterygoid process. The bone was now readily removed by dividing the soft parts with the bistoury and scissors. Several spiculæ of bone were removed with the forceps and bone nippers. All the arteries of any size were ligated; the hemorrhage from the smaller vessels and capillaries was arrested by the application of the perchloride of iron. After filling the space occupied by the bone with lint, the edges of the two flaps were brought together, as in the first operation, with delicate pins, and the same dressing adopted. Stimulants were administered several times during the operation, and once or twice the operation was suspended for a few minutes that the patient might have some repose, which in both instances acted happily in restoring to some extent the force of the circulation. At six o'clock, P. M., the patient was put to bed, and a half grain of the sulphate of morphine and two ounces of brandy administered. At nine o'clock, three hours afterwards, I saw the patient; no hemorrhage; pulse less frequent; more comfortable; half grain morphine was again administered, with instructions to repeat it if there was much restlessness during the night.

Dec. 16th, six o'clock, A. M.—Reaction almost perfect; no hemorrhage; slept some during the night; appears comfortable.

One o'clock, P. M.—Reaction perfect; pulse 100, and sufficiently full; considerable pain in the left side of the face and head; ordered half grain morphine.

Eight o'clock, P. M.—Pulse 110; comfortable; ordered an anodyne to be given, if necessary.

Dec. 17th, eight o'clock, A. M.—Passed rather a comfortable night; pulse 95; has taken a little soup; considerable tumefaction about the face; but little pain.

Ten o'clock, P. M.—Pulse 98; doing well; ordered an anodyne, if necessary.

Dec. 18th, ten o'clock, A. M.—Passed an uncomfortable night; feels much more pleasant this morning; pulse 90; removed every alternate pin.

Dec. 19.—Removed all the remaining pins except the one at the commissure of the lips; union by the first intention had taken place for a considerable extent; several strips of adhesive plaster were applied to support the part.

After this the patient continued to improve, nothing occurring worth noting. I neglected to mention that the lint introduced immediately after the operation was removed on the second day, and the parts cleansed with tepid water and a syringe, and more lint introduced. This was done every day or every second day, for the first eight or ten days.

At the expiration of fifteen days, the wound had entirely healed, and the patient was able to walk about the room. The tumefaction had not yet subsided, but had greatly diminished. Two weeks after, making four weeks after the operation, the patient left for home, without any appearance of a return of the disease.

I fear, however, that the disease will some day re-appear, and if so, must necessarily prove fatal in a very short time.

